# Randomized trial of radiofrequency ablation *versus* conventional surgery for superficial venous insufficiency: if you don’t tell, they won’t know

**DOI:** 10.6061/clinics/2016(11)06

**Published:** 2016-11

**Authors:** Cynthia de Almeida Mendes, Alexandre de Arruda Martins, Juliana Maria Fukuda, José Ben-Hur Ferraz Parente, Marco Antonio Soares Munia, Alexandre Fioranelli, Marcelo Passos Teivelis, Andrea Yasbek Monteiro Varella, Roberto Augusto Caffaro, Sergio Kuzniec, Nelson Wolosker

**Affiliations:** I Hospital Israelita Albert Einstein, Division of Vascular and Endovascular Surgery, São Paulo/SP, Brazil; II Irmandade da Santa Casa de Misericórdia de São Paulo, Division of Vascular and Endovascular Surgery, São Paulo/SP, Brazil; III Hospital Municipal Dr. Moysés Deutsch – M’Boi Mirim, São Paulo/SP, Brazil

**Keywords:** Chronic Venous Insufficiency, Great Saphenous Vein, Radiofrequency Ablation, Surgery

## Abstract

**OBJECTIVES:**

This study compared radiofrequency ablation *versus* conventional surgery in patients who had not undergone previous treatment for bilateral great saphenous vein insufficiency, with each patient serving as his own control.

**METHOD:**

This was a randomized controlled trial that included 18 patients and was carried out between November 2013 and May 2015. Each of the lower limbs of each patient was randomly assigned to undergo either radiofrequency ablation or conventional surgery. Clinical features (hyperpigmentation, hematoma, aesthetics, pain, skin burn, nerve injury, and thrombophlebitis) were evaluated at one week, one month, and six months postoperatively. Hemodynamic assessments (presence of resection or occlusion of the great saphenous vein and recurrent reflux in the sapheno-femoral junction and in the great saphenous vein) were performed at one month, six months, and 12 months postoperatively. The independent observer (a physician not involved in the original operation), patient, and duplex ultrasonographer were not made aware of the treatment done in each case. Clinicaltrials.gov: NCT02588911.

**RESULTS:**

Among the clinical variables analyzed, only the aesthetic evaluation by the physicians was significant, with radiofrequency ablation being considered better than conventional surgery (average, 0.91 points higher: standard deviation: 0.31; 95% confidence interval: -1.51, -0.30; *p*=0.003). However, in our study, we observed primary success rates of 80% for radiofrequency ablation and 100% for conventional surgery.

**CONCLUSIONS:**

If the physician is not required to inform the patient as to the technique being performed, the patient will not be able to identify the technique based on the signs and symptoms. Our study revealed that both techniques led to high levels of patient satisfaction, but our results favor the choice of conventional surgery over radiofrequency ablation, as patients who underwent conventional surgery had better hemodynamic assessments.

## INTRODUCTION

Superficial venous insufficiency (VI), which affects millions of patients worldwide, is one of the most common conditions observed by vascular surgeons in clinical practice. The prevalence of superficial VI varies greatly and is highest among Western populations, with varicose veins (CEAP 2) being observed in 13% to 46% of women and 11% to 29% of men 1,2. Superficial VI can cause leg fatigue, pain and swelling and can lead to more serious complications such as deep vein thrombosis (DVT) and stasis ulcers. Thrombophlebitis, lipodermatosclerosis, and bleeding veins have also been reported to be associated with superficial VI. Most cases of varicose veins (70%) are due to sapheno-femoral junction (SFJ) insufficiency and/or great saphenous vein (GSV) reflux. The extent of saphenous vein reflux is directly correlated with disease symptoms 3.

Due to its progressive nature, superficial VI can cause increasingly debilitating symptoms as the patient ages, especially if left untreated. In addition to having a considerable impact on the patient’s quality of life, superficial VI is a major public health concern, as it may result in the loss of working days and high costs for the local health system (both public and private) 4.

Treatment of superficial VI may be interventional or supportive. Interventional treatments include the following: 1) conventional surgery (CS), 2) thermal ablation techniques such as radiofrequency ablation (RFA) and endovenous laser ablation (EVLA), and 3) ultrasound-guided foam sclerotherapy (UGFS). Supportive treatments include compression stockings 5-8. Interventional procedures can be performed on an inpatient or outpatient basis, with the latter option being associated with lower costs 9,10.

CS and thermal ablation approaches are considered the best forms of treatment for this progressive disease. Until recently, CS was the gold standard treatment for GSV insufficiency, with good initial success rates and low recurrence rates in the short term 11,12. The more recently developed thermal ablation techniques, such as RFA and EVLA, involve catheter-based ablation of the GSV. Studies reporting the safety of these techniques also reported higher recurrence rates after endovenous techniques 13,14.

Postoperatively, variables such as recovery time, complications, aesthetic results, time away from work, and costs vary among the reports, and whether RFA or EVLA is advantageous relative to CS in terms of these variables is still controversial 1.

A meta-analysis of 28 randomized controlled trials (RCTs) revealed that primary failure and recurrence did not differ significantly by technique (EVLA, RFA, and CS). However, these thermal ablation techniques resulted in fewer hematomas and wound infections, as well as less pain and a quicker return to normal activities 15. This meta-analysis included studies that allocated each patient to a different technique as well as studies that applied more than one technique in a single patient (one in each leg). In the only study that directly compared RFA and CS in the same patient, the patients had undergone a previous SFJ ligation that resulted in the recurrence of GSV insufficiency 16.

In the current study, we conducted an RCT in which we randomized the lower limbs of each patient to RFA or CS. The patients had no history of previous treatment for bilateral GSV insufficiency. Each leg was assessed for postoperative symptoms and complications. In addition, duplex ultrasound was used to evaluate the presence of resection or occlusion of the GSV, as well as reflux in the SFJ and GSV. To the best of our knowledge, no study to date has compared RFA versus CS in patients who served as their own controls and who had intact GSVs.

## MATERIALS AND METHODS

The study was carried out in the Department of Vascular Surgery of a secondary referral hospital between November 2013 and May 2015.

The inclusion and exclusion criteria are summarized in Table 1. A total of 18 patients entered the trial, which consisted of a randomized controlled study. According to the protocol, each patient was treated with RFA on one leg and CS on the contralateral limb. Randomization was performed preoperatively using a randomization table. Patients were not advised of the treatment allocation to ensure that this trial was carried out in a blinded fashion. All operations were performed under regional anesthesia administered via spinal block by the same surgical team who was skilled in the management of venous disease and had extensive expertise in both techniques. Phlebectomy of varicosities and treatment of incompetent perforating veins were not concomitantly performed.

### CS

Patients underwent a standard procedure of cranial ligation of the GSV and branches of the SFJ using a groin crease incision and stripping of the GSV from the SFJ to ankle level using a vein stripper that was extracted through a small incision near the medial malleolus.

### RFA

The procedure was performed under ultrasound guidance. The GSV proximal to the medial malleolus was cannulated with a 7F sheath using the surgical cutdown approach. The tip of the radiofrequency catheter was placed at least 2 cm distal to the SFJ or just distal to the superficial epigastric vein orifice. Patients received tumescent infiltration with cold normal saline (0.9%) circumferentially around the GSV within its enveloping fascia and along the entire length of the treated vein; this was to prevent nerve injury and thermal injury to the skin. Then, the catheter was slowly withdrawn according to the device manufacturer’s recommendations. The technique consisted of controlled segmental heating of the GSV using a catheter with a 7-cm heating element (Closure**™** system, VNUS Medical Technologies, Inc., San Jose, California, USA), followed by manual compression over the GSV. The temperature was maintained at 120°C per segment for a standard length of time. The temperature-controlled RFA continued until the catheter tip reached just below the knee. Immediately following treatment with RFA, intraoperative ultrasound imaging was used to confirm shrinkage of the vein.

To ensure that the independent observer, a physician who was responsible for outcome assessment and not involved in the original operation and the patient were not made aware of the treatment performed in each case, a groin crease incision and an incision proximal to the medial malleolus were created on both legs. For limbs operated on using the radiofrequency technique, a groin crease incision was made similar to the contralateral side but with no manipulation of the SFJ. The incision proximal to the medial malleolus was used for sheath insertion.

Postoperatively, dressings were placed over the wounds and the patients’ legs were wrapped in sterile absorbent bandages and covered with a cohesive compression bandage for 48 hours. Patients were instructed to immediately lie down with their legs elevated and to walk for progressively longer periods each day. After removal of the bandages, patients were instructed to use 20- to 30-mmHg compression stockings for four weeks. All patients were discharged on the same day as the procedure and were encouraged to resume work and normal activity as soon as they were able. Analgesics and nonsteroidal anti-inflammatory drugs were prescribed to the patients.

There were 18 patients (36 legs), including 11 women and 7 men. The average age was 48.1 years (range: 33-76; standard deviation (SD): 12.7). The mean body mass index was 28.1 kg/m^2^ (range: 22.4-34.9; SD: 3.5). There were no diabetic patients; only three patients had a positive history of smoking, and only six patients had high blood pressure.

For clinical assessments, patients were scheduled for follow-up visits at one week, one month, and six months after surgery. For duplex ultrasound scan assessments, patients were scheduled for follow-up visits at one month, six months, and 12 months after surgery. Twelve patients were followed for up to one year and the remaining six patients were followed for six months.

Each leg was assessed for postoperative symptoms and complications by an independent observer, who was a physician not involved in the original operation and by the patient. The surgeons were not involved in the outcome assessments. The clinical outcomes included intensity of hyperpigmentation, extension of hematoma, aesthetic results, pain levels, severity of skin burns, nerve injury, and thrombophlebitis. Patients and physicians were instructed to indicate their subjective perception of hyperpigmentation and hematomas on a scale of 0 (no complaint/discomfort) to 10 (maximal complaint/discomfort) as well as aesthetic results on a scale of 0 (unaesthetic) to 10 (excellent results). Patients were also asked to indicate their pain levels on a scale of 0 (no pain) to 10 (worst imaginable pain) and to indicate any changes in sensitivity. Physicians were also asked to indicate the severity of skin burns on a scale of 0 (no skin burns) to 10 (severe skin burns) and to report the presence of thrombophlebitis.

Duplex ultrasonography was used to evaluate the following hemodynamic outcomes: the presence of resection or occlusion (success rate) of the GSV and reflux in the SFJ and GSV, as well as the presence of complications such as DVT and lymphocele. The duplex ultrasonographer was not made aware of the treatment performed on each side before examining the leg.

### Statistical analysis

Regarding demographic characteristics, the quantitative variables are reported as the mean ± standard deviation, and the categorical variables are reported as the absolute and relative frequencies.

The hemodynamic outcomes are presented as the duplex ultrasound scan findings and moments of evaluation using absolute and relative frequencies. However, a statistical analysis could not be performed because the sample size of 18 was too small.

The clinical outcomes are described as types of technique and moments of evaluation and are reported as the mean ± standard deviation. The variables were compared between techniques and moments of evaluation using generalized estimating equations with an exchangeable correlation matrix and assuming a normal distribution with an identity link function. For models with statistical significance, an additional analysis using the Bonferroni multiple comparison test was performed.

A probability value (*p* value) of less than 0.05 was considered statistically significant. The 95% confidence interval (CI) was also included where appropriate.

For our analyses, we calculated three *p* values: one corresponding to the technique across all three moments of evaluation (p_technique_); one corresponding to the moment of evaluation (which considered both techniques together, p_moment_); and one corresponding to the interaction between technique and moment (p_interaction_).

### Ethics

Approval was obtained from the Local Research Ethics Committee (Plataforma Brasil CAAE03772812.7.0000.0071), and all patients provided written informed consent for inclusion in the trial.

## RESULTS

Hyperpigmentation, hematoma, pain, aesthetic perception, thrombophlebitis, nerve injury, and skin burns were assessed after surgery. Of these variables, thrombophlebitis, nerve injury, and skin burns exhibited null results. Subjective ratings for the first four variables are summarized in Tables 2 and 3.

The subjective assessments conducted by patients and physicians are presented in Table 2. The p_interaction_ result was not statistically significant for any of the variables studied, indicating that the difference between techniques (RFA and CS) did not vary over time. The only variable that reached significance when comparing both techniques was aesthetic evaluation by physicians, which revealed that the RFA-treated limb received, on average, an evaluation of 0.91 points higher (SD: 0.31; 95% CI: -1.51, -0.30; p_technique_=0.003) than the conventionally treated side. At six months, the physicians assigned 10 points each to all the limbs in the aesthetic evaluation. There were significant differences between the moments in both the physicians’ and patients’ evaluations of hematoma and aesthetics and in the patients’ evaluation of pain (p_moment_).

The comparisons between the different moments studied are presented in Table 3. For p_moment_, we observed that the perception of the extension of hematoma was lower at one and six months compared to one week for both physicians and patients (*p*<0.001). However, the perception at one month was not significantly different from that at six months for either technique. For both techniques, the aesthetic evaluation progressively improved over time (*p*<0.05). Finally, pain level progressively and significantly decreased from one week to one month and six months (*p*<0.05).

There were no minor or major postoperative complications. Importantly, no cases of DVT were observed during follow-up. All patients took less than one week to return to work and used compression stockings for the recommended length of time.

Immediate intra-operative success was reported in all cases, with complete resection or occlusion of the GSV.

For limbs operated on using the conventional technique, there were no cases of reflux in the SFJ and no cases of segmental reflux at any time. For limbs operated on using the radiofrequency technique, the closure rate improved over time, but only 80% of the GSVs studied exhibited complete obliteration after one year. Reflux involving the SFJ and GSV was observed in one patient on the 30th day; however, this resolved over time, as shown by the six-month duplex ultrasound scan exam (Table 4).

## DISCUSSION

Among the clinical variables analyzed, only the aesthetic evaluation by physicians reached significance, with RFA being considered better than CS. On the other hand, CS had better results due to its superior success rate.

For the patients, the aesthetic evaluation did not differ by technique. This conclusion may reflect the fact that physicians tend to overestimate the postoperative results, as they have more experience in evaluating clinical outcomes, whereas patients provide their perception based on their personal life experiences.

Our study identified primary success rates of 80% for RFA and 100% for CS. These rates are comparable to those in previous reports 17-19.

Following CS, recurrence may be related to technical or tactical failure, an incompetent below-knee GSV, neovascularization leading to neoreflux in the groin, and new incompetent perforators; however, considering RFA, recurrence may be attributable to an incompetent below-knee GSV, disease progression with neoreflux in the groin tributaries, and recanalization of a previously occluded GSV 1.

In the present study, a groin incision without tissue dissection was made on limbs operated on using the radiofrequency technique. Some authors have suggested that patients undergoing RFA may be less prone to neovascularization because the SFJ is left untouched 20. Neovascularization is thought to be the consequence of angiogenesis following tissue trauma due to surgical dissection, and it has been implicated as the main cause of recurrence in several studies 21,22. This is ultimately linked to the need for reintervention, which is a cause of patient dissatisfaction with the technique 23.

It has been suggested that the observed high recurrence rates following CS are related to the technical inadequacy of the initial procedure 24. A larger number of recurrences has been associated with insufficiency in the below-knee GSV after stripping of the GSV to the knee only 25. It has been argued that the length of stripping should be dictated by the length of the refluxing vein and not by concerns over injury to the saphenous nerve 26. The superior success rate reported for CS in our study can be explained by the fact that we performed stripping of the GSV from the SFJ to the ankle level. However, full-length stripping remains controversial.

Although the issues of recurrence and neovascularization are important, our trial was not designed to study them in depth. Furthermore, the sample size was too small to draw definitive conclusions regarding the clinical results of recanalized segments of the GSV.

The design of the present study to compare the two different techniques in the same patient undergoing treatment for GSV reflux has been used previously 27,28 but not in patients without a history of previous treatment for bilateral GSV insufficiency. In the only previous study that directly compared RFA and CS in the same patient, 16 patients had undergone a previous SFJ ligation that resulted in the recurrence of GSV insufficiency. In that study, the authors observed that RFA treatment was faster and resulted in less pain and bruising. The complete success rates were 81.2% and 87.5% for RFA and CS, respectively, as two GSVs were partially stripped in the limbs operated on using CS. The maximum follow-up was 12 months 16.

Most of the available trials compare different techniques performed in different patients. For this reason, the results of these studies cannot be directly compared to our results. RFA has been shown to be superior to CS in terms of short-term and medium-term outcomes such as pain, return to activities, quality of life, and patient satisfaction 19,29. One RCT that randomized patients to RFA or CS revealed that RFA was more expensive, but it enabled patients to return to work one week earlier than after CS; patients were followed up toward the end of the first and fifth weeks after surgery 18. Another study that compared RFA and CS showed that RFA was more expensive, had a lower overall complication rate (including pain and hematoma) and had a shorter post-intervention hospital stay; the follow-up visits ranged from six months to two years after intervention 17.

Our results indicated that regardless of the technique performed, patients’ evaluations of hematoma, aesthetics, and pain improved over time, indicating that the patients were more satisfied at six months than at one week. In our study, none of the patients developed DVT, thrombophlebitis, nerve injury, or skin burns. Considering DVT, previous studies reported comparable rates of 1% 30, 0% 31, and 0% 17. Neurological damage is among the most common side effects of GSV stripping. Saphenous nerve injury occurs in approximately 40% of cases of long stripping of the GSV but with little significant morbidity 32.

The sham incision was part of the protocol design for the express purpose of not allowing the patient or the observer (physician) to be aware of the technique used on each limb. From a methodological point of view, this was the only viable approach to ensure real blinding. The body of literature related to the issue of using a sham incision reveals that it is considered ethical by many authors 33,34.

We emphasized to all of the patients that such a procedure could be associated with minimal risks (as observed in our study, in which there were no complications related to groin incisions, which were only necessary to ensure scientific accuracy) and would only be performed in those patients who had agreed to the study and had signed the informed consent form. Patients were given the option of not participating in the research study and being treated with standard therapy.

As previously mentioned, our study received approval from the Local Research Ethics Committee. The Local Research Ethics Committee understood that there would be minimal risks involved and that the patients were free to refuse to participate in the research and to instead be treated with standard therapy.

As the number of collateral veins and perforators may vary significantly between one leg and the contralateral leg, phlebectomy of varicosities and treatment of incompetent perforating veins were not concomitantly performed to avoid having an impact as a confounding variable on the evaluation of the extension of hematoma and pain levels 35. It is possible that lower limbs with more avulsions had more bruising and pain.

In our study, it was not possible to analyze and compare quality of life, return to normal activities, loss of productivity due to sickness leave, detailed costs related to the type of anesthesia used (local *vs*. regional), the choice of treatment system (outpatient *vs*. inpatient), and the use of specialized equipment, as both techniques were conducted in the same individual.

Another limitation of our study was the loss to follow-up over time, as an analysis of all variables in the complete sample could have increased the statistical significance. Our trial was not specifically designed to assess long-term outcomes. Further follow-up will hopefully allow robust conclusions to be drawn.

The most significant aspect of our trial was the comparison of CS and RFA using a study design in which the patients served as their own controls. lf the physician is not required to inform the patient as to the technique being performed, the patient will not be able to identify the technique based on the signs and symptoms. Our study revealed that both techniques led to high levels of patient satisfaction, but our results favor the choice of CS over RFA, as patients who underwent CS had better hemodynamic assessments.

## AUTHOR CONTRIBUTIONS

Mendes CA designed the study, analyzed the data and drafted the manuscript. Martins AA designed the study and drafted the manuscript. Fukuda JM analyzed the data and drafted the manuscript. Parente JB and Munia MA designed the study and drafted the manuscript. Fioranelli A designed the study and analyzed the data. Teivelis MP and Varella AY analyzed the data and drafted the manuscript. Caffaro RA and Kuzniec S designed the study and analyzed the data. Wolosker N designed the study, analyzed the data and drafted the manuscript.

## Figures and Tables

**Table 1:** Inclusion and exclusion criteria.

Inclusion criteria	Exclusion criteria
Age between 18 and 60 years Clinical, etiological, anatomical, pathophysiological (CEAP): clinical grades 2 to 5 (C2-5), primary (Ep), superficial (As), and reflux only (Pr) Primary bilateral GSV insufficiency requiring surgery and confirmed by duplex scan (insufficiency with reverse venous flow was regarded significant if persisting more than 0.5 seconds in a standing position) Suitability for radiofrequency ablation confirmed by duplex scan (see exclusion criteria) Patients able to give informed consent	Varicose veins without GSV insufficiency on duplex scan Previous varicose vein surgery Associated small saphenous vein reflux, duplication of the GSV at the SFJ, deep venous insufficiency, or previous deep vein thrombosis on duplex scan GSV diameter <3 mm or >12 mm in the supine position Thrombus in the GSV Patients with a pacemaker or internal defibrillator Concomitant peripheral arterial disease (ankle-brachial pressure index of <0.9) Patients on oral anticoagulants Patients with high blood pressure not controlled by medication Patients with known thrombophilia, cancer or lupus Pregnancy

GSV, great saphenous vein; SFJ, sapheno-femoral junction.

**Table 2:** Subjective assessments conducted by patients and physicians of the surgical techniques over time.

	Moment	Conventional Surgery Mean±SD	Radiofrequency Ablation Mean±SD	p_technique_	p_moment_	p_interaction_
Hyperpigmentation (physician)	1 week	0.00±0.00	0.00±0.00	0.644	0.179	0.802
	1 month	0.24±0.97	0.12±0.49			
	6 months	0.00±0.00	0.00±0.00			
Hyperpigmentation (patient)	1 week	0.00±0.00	0.00±0.00	0.348	0.403	0.403
	1 month	0.47±1.94	0.00±0.00			
	6 months	0.00±0.00	0.00±0.00			
Hematoma (physician)	1 week	4.86±2.93	4.21±3.07	0.194	<0.001	0.598
	1 month	1.00±1.95	0.00±0.00			
	6 months	0.00±0.00	0.00±0.00			
Hematoma (patient)	1 week	4.50±2.79	4.21±3.60	0.483	<0.001	0.837
	1 month	0.45±1.04	0.00±0.00			
	6 months	0.00±0.00	0.00±0.00			
Aesthetic evaluation (physician)	1 week	6.29±2.02	7.43±1.79	0.003	<0.001	0.089
	1 month	7.27±1.79	8.82±1.47			
	6 months	10.00±0.00	10.00±0.00			
Aesthetic evaluation (patient)	1 week	6.93±2.70	6.86±2.71	0.843	<0.001	0.936
	1 month	7.45±3.88	7.82±3.03			
	6 months	10.00±0.00	10.00±0.00			
Pain	1 week	5.64±3.80	3.71±3.27	0.060	<0.001	0.309
	1 month	2.14±2.74	1.08±2.07			
	6 months	0.00±0.00	0.00±0.00			

SD, standard deviation

For our analyses, we calculated three p values: one corresponding to the technique (p_technique_), one corresponding to the evaluation moment (p_moment_), and one corresponding to the interaction between technique and moment (p_interaction_).

**Table 3:** Comparison between the different moments studied for hematoma, aesthetic result, and pain.

	Comparisons	Mean difference±SD	95% CI	*p*
Hematoma (physician)	1 week *vs* 1 month	4.16±0.56	2.83 to 5.49	<0.001
	1 week *vs* 6 months	4.58±0.52	3.33 to 5.82	<0.001
	1 month *vs* 6 months	0.42±0.55	-0.90 to 1.74	>0.999
Hematoma (patient)	1 week *vs* 1 month	4.16±0.56	2.81 to 5.51	<0.001
	1 week *vs* 6 months	4.36±0.53	3.10 to 5.62	<0.001
	1 month *vs* 6 months	0.20±0.56	-1.15 to 1.54	>0.999
Aesthetic evaluation (physician)	1 week *vs* 1 month	-1.16±0.39	-2.09 to -0.22	0.009
	1 week *vs* 6 months	-3.13±0.36	-3.99 to -2.27	<0.001
	1 month *vs* 6 months	-1.97±0.38	-2.89 to -1.05	<0.001
Aesthetic evaluation (patient)	1 week *vs* 1 month	-0.56±0.65	-2.10 to 0.99	>0.999
	1 week *vs* 6 months	-3.07±0.59	-4.48 to -1.65	<0.001
	1 month *vs* 6 months	-2.51±0.63	-4.02 to -1.00	<0.001
Pain	1 week *vs* 1 month	3.06±0.66	1.48 to 4.64	<0.001
	1 week *vs* 6 months	4.70±0.64	3.18 to 6.22	<0.001
	1 month *vs* 6 months	1.64±0.65	0.08 to 3.19	0.035

CI, confidence interval; SD, standard deviation.

**Table 4:** For limbs operated on using the radiofrequency technique: the number of patients showing venous occlusion or reflux at each of the time points studied as assessed by duplex ultrasound scan and the number of patients with each condition out of the total number of patients assessed (%).

	At day 30	At 6 months	At 12 months
Complete occlusion of the great saphenous vein	13/17 (76.5)	12/15 (80.0)	8/10 (80.0)
Reflux in the sapheno-femoral junction	1/17 (5.9)	0/15 (0.0)	0/10 (0.0)
Reflux in the great saphenous vein	1/17 (5.9)	0/15 (0.0)	0/10 (0.0)

## References

[1] Wittens C, Davies AH, Bækgaard N, Broholm R, Cavezzi A, Chastanet S (2015). Editor's Choice - Management of Chronic Venous Disease: Clinical Practice Guidelines of the European Society for Vascular Surgery (ESVS). Eur J Vasc Endovasc Surg.

[2] Campbell B (2014). New evidence on treatments for varicose veins. Br J Surg.

[3] Ruckley CV, Evans CJ, Allan PL, Lee AJ, Fowkes FG (2002). Chronic venous insufficiency: clinical and duplex correlations. The Edinburgh Vein Study of venous disorders in the general population. J Vasc Surg.

[4] Carroll C, Hummel S, Leaviss J, Ren S, Stevens JW, Everson-Hock E (2013). Clinical effectiveness and cost-effectiveness of minimally invasive techniques to manage varicose veins: a systematic review and economic evaluation. Health Technol Assess.

[5] Perkins JM (2009). Standard varicose vein surgery. Phlebology.

[6] Gohel MS, Davies AH (2009). Radiofrequency ablation for uncomplicated varicose veins. Phlebology.

[7] Darwood RJ, Gough MJ (2009). Endovenous laser treatment for uncomplicated varicose veins. Phlebology.

[8] Coleridge Smith P (2009). Foam and liquid sclerotherapy for varicose veins. Phlebology.

[9] Eidson JL, Atkins MD, Bohannon WT, Marrocco CJ, Buckley CJ, Bush RL (2011). Economic and outcomes-based analysis of the care of symptomatic varicose veins. J Surg Res.

[10] Gohel MS, Epstein DM, Davies AH (2010). Cost-effectiveness of traditional and endovenous treatments for varicose veins. Br J Surg.

[11] Sarin S, Scurr JH, Coleridge Smith PD (1992). Assessment of stripping the long saphenous vein in the treatment of primary varicose veins. Br J Surg.

[12] Dwerryhouse S, Davies B, Harradine K, Earnshaw JJ (1999). Stripping the long saphenous vein reduces the rate of reoperation for recurrent varicose veins: five-year results of a randomized trial. J Vasc Surg.

[13] Christenson JT, Gueddi S, Gemayel G, Bounameaux H (2010). Prospective randomized trial comparing endovenous laser ablation and surgery for treatment of primary great saphenous varicose veins with a 2-year follow-up. J Vasc Surg.

[14] Perälä J, Rautio T, Biancari F, Ohtonen P, Wiik H, Heikkinen T (2005). Radiofrequency endovenous obliteration versus stripping of the long saphenous vein in the management of primary varicose veins: 3-year outcome of a randomized study. Ann Vasc Surg.

[15] Siribumrungwong B, Noorit P, Wilasrusmee C, Attia J, Thakkinstian A (2012). A systematic review and meta-analysis of randomised controlled trials comparing endovenous ablation and surgical intervention in patients with varicose vein. Eur J Vasc Endovasc Surg.

[16] Hinchliffe RJ, Ubhi J, Beech A, Ellison J, Braithwaite BD (2006). A prospective randomised controlled trial of VNUS closure versus surgery for the treatment of recurrent long saphenous varicose veins. Eur J Vasc Endovasc Surg.

[17] Helmy ElKaffas K, ElKashef O, ElBaz W (2011). Great saphenous vein radiofrequency ablation versus standard stripping in the management of primary varicose veins-a randomized clinical trial. Angiology.

[18] Subramonia S, Lees T (2010). Radiofrequency ablation vs conventional surgery for varicose veins - a comparison of treatment costs in a randomised trial. Eur J Vasc Endovasc Surg.

[19] Lurie F, Creton D, Eklof B, Kabnick LS, Kistner RL, Pichot O (2003). Prospective randomized study of endovenous radiofrequency obliteration (closure procedure) versus ligation and stripping in a selected patient population (EVOLVeS Study). J Vasc Surg.

[20] Pichot O, Kabnick LS, Creton D, Merchant RF, Schuller-Petroviae S, Chandler JG (2004). Duplex ultrasound scan findings two years after great saphenous vein radiofrequency endovenous obliteration. J Vasc Surg.

[21] Jones L, Braithwaite BD, Selwyn D, Cooke S, Earnshaw JJ (1996). Neovascularisation is the principal cause of varicose vein recurrence: results of a randomised trial of stripping the long saphenous vein. Eur J Vasc Endovasc Surg.

[22] Nyamekye I, Shephard NA, Davies B, Heather BP, Earnshaw JJ (1998). Clinicopathological evidence that neovascularisation is a cause of recurrent varicose veins. Eur J Vasc Endovasc Surg.

[23] Winterborn RJ, Foy C, Earnshaw JJ (2004). Causes of varicose vein recurrence: late results of a randomized controlled trial of stripping the long saphenous vein. J Vasc Surg.

[24] Egan B, Donnelly M, Bresnihan M, Tierney S, Feeley M (2006). Neovascularization: an “innocent bystander” in recurrent varicose veins. J Vasc Surg.

[25] Joshi D, Sinclair A, Tsui J, Sarin S (2011). Incomplete removal of great saphenous vein is the most common cause for recurrent varicose veins. Angiology.

[26] Kostas TT, Ioannou CV, Veligrantakis M, Pagonidis C, Katsamouris AN (2007). The appropriate length of great saphenous vein stripping should be based on the extent of reflux and not on the intent to avoid saphenous nerve injury. J Vasc Surg.

[27] de Medeiros CA (2006). Comparison of endovenous laser therapy vs. conventional stripping of the great saphenous vein: midterm results. J Vasc Bras.

[28] Goode SD, Chowdhury A, Crockett M, Beech A, Simpson R, Richards T (2010). Laser and radiofrequency ablation study (LARA study): a randomised study comparing radiofrequency ablation and endovenous laser ablation (810 nm). Eur J Vasc Endovasc Surg.

[29] Merchant RF, Pichot O, Closure Study Group (2005). Long-term outcomes of endovenous radiofrequency obliteration of saphenous reflux as a treatment for superficial venous insufficiency. J Vasc Surg.

[30] Merchant RF, DePalma RG, Kabnick LS (2002). Endovascular obliteration of saphenous reflux: a multicenter study. J Vasc Surg.

[31] Weiss RA, Weiss MA (2002). Controlled radiofrequency endovenous occlusion using a unique radiofrequency catheter under duplex guidance to eliminate saphenous varicose vein reflux: a 2-year follow-up. Dermatol Surg.

[32] Morrison C, Dalsing MC (2003). Signs and symptoms of saphenous nerve injury after greater saphenous vein stripping: prevalence, severity, and relevance for modern practice. J Vasc Surg.

[33] Miller FG (2004). Sham surgery: an ethical analysis. Sci Eng Ethics.

[34] Wolf BR, Buckwalter JA (2006). Randomized surgical trials and “sham” surgery: relevance to modern orthopaedics and minimally invasive surgery. Iowa Orthop J.

[35] Welch HJ (2006). Endovenous ablation of the great saphenous vein may avert phlebectomy for branch varicose veins. J Vasc Surg.

